# Life threatening hyperkalemia following cocaine ingestion: a case report

**DOI:** 10.4076/1757-1626-2-7355

**Published:** 2009-07-30

**Authors:** Faisal Siddiqui, Roger Slater, Sara Ashraf

**Affiliations:** 1 Department of Anaesthetics, Central Manchester & Manchester Childrens University Hospitals NHS TrustOxford Road, Manchester, M13 9WLUK; 2 Department of Emergency Medicine, University Hospital of South ManchesterSouthmoor Road Wythenshawe, M23 9LTUK

## Abstract

We present a case of life-threatening hyperkalemia after recreational cocaine ingestion. Acute cocaine-induced rhabdomyolysis led to hyperkalemia, cardiac arrhythmias and cardiogenic shock resulting in multi-organ failure.

## Case presentation

**Figure 1.**
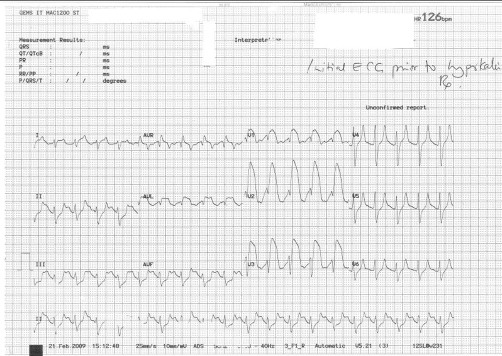
Initial ECG prior to hyperkalemia treatment reveals wide QRS complexes, Tall peaked T waves and ST segment elevation.

**Figure 2.**
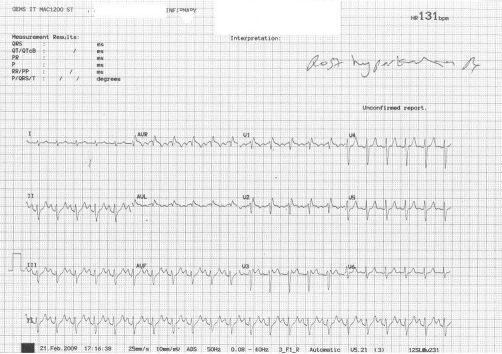
Reversal to normal sinus rhythm after hyperkalemia treatment. T waves reverted back to normal morphology.

A 26-year-old Caucasian man of British ethnicity was brought to the emergency department having been found unconscious at home in bed. There was a history of cocaine and alcohol ingestion the previous evening. The exact timing of his collapsed state in relation to the cocaine ingestion was unclear. His Glasgow Coma Score on arrival was 4. He was hypoxic (oxygen saturation 80%), with a respiratory rate of 50 breaths/min, heart rate of 120 and blood pressure of 90/43. Initial management consisted of oxygen and rapid fluid infusion. He required a rapid sequence induction, endotracheal intubation and mechanical ventilation. The ECG was interpreted as showing a broad complex tachycardia (Figure 1). Initial blood gas showed a PaO_2_ 8.8 KPa, PaCO_2_ 7.52 KPa, pH 7.0 and base excess was -12 with a serum lactate of 7.2. Biochemistry revealed a serum potassium 8.9 of mmol/l, Alanine Transaminase of ~6500, Creatinine of 395, Troponin of 0.7 and creatinine kinase was unrecordably high. He was given insulin and dextrose (10 units in 50 ml 50% glucose) to treat the hyperkalemia followed by synchronized cardioversion (50 J, 100 J × 2) in an attempt to restore sinus rhythm. He remained hypotensive and was therefore commenced on a dobutamine infusion in the emergency department. Initial echocardiogram revealed severe systolic dysfunction with an ejection fraction of 20%. Because of the high risk of coronary vasospasm from cocaine and a strong family history of ischaemic heart disease he was taken for urgent coronary angiography. However the coronary vasculature was normal. Computed tomography of the brain was also normal. His cardiac rhythm normalised over the next 2 hours with a sinus tachycardia once the serum potassium levels were corrected to 5.1 mmol/l (Figure 2). He remained intubated and ventilated and was transferred to intensive care. His oxygenation rapidly deteriorated and a chest X-ray showed a bat’s-wing appearance of pulmonary oedema. The metabolic acidosis persisted and he was noted to be anuric. The working diagnosis was rhabdomyolysis leading to renal failure and hyperkalemia. He required prone ventilation and continuous haemofiltration. Over the next 8 days his condition slowly improved and following a tracheostomy he was successfully weaned from ventilatory support but continued to require intermittent haemodialysis. He was discharged to the ward but 2 weeks later he suffered a grand mal convulsion and was found to have bilateral basal ganglia and internal capsule infarcts possibly secondary to the cocaine ingestion.

## Discussion

Cocaine is a popular drug of abuse and there is a tremendous surge in its use because of its stimulant and euphoric properties [1]. It has multi-systemic involvement and there are many reports detailing its harmful effects. Cocaine toxicity normally occurs in chronic abusers and is mainly due to excessive central nervous system stimulation and excessive adrenergic vasoconstriction, the latter resulting in severe hypertension and/or organ ischaemia with associated organ injury [2]. Rhabdomyolysis is a recognised complication of cocaine toxicity [3]. It has been postulated that this results from a combination of sympathetic and muscular overactivity leading to vasoconstriction, micro-infarcts and skeletal muscle necrosis and local pressure necrosis whilst the patient is obtunded [4]. Cocaine-induced hyperpyrexia with fluid loss and inadequate replacement may also precipitate acute renal failure. The cardiovascular complications of cocaine abuse are adrenergic mediated and range from cocaine-associated acute coronary syndromes to aortic dissection and sudden cardiac death [5]. Mortality secondary to cocaine abuse is usually not secondary to single drug overdose but most deaths occur after prolonged drug use, which initiates a series of changes at the molecular, cellular, and tissue levels [6]. All of these changes favour sudden death [6]. Additionally, cocaine use has been associated with spontaneous coronary dissection, mesenteric ischemia, stroke, venous thrombosis, and a variety of pulmonary complications [7].

Wide complex dysrhythmia due to cocaine in the absence of myocardial Infarction, as happened in our case, is rare and optimum management is undefined [8]. It was postulated that this was due to direct myocardial sodium channel antagonism similar to class I antidysrhythmic drugs and is treated by administration of IV Sodium Bicarbonate. Cocaine ingestion can also lead to Brugada electrocardiographic pattern and can be misleading as it is similar to the ST elevation that occurs in cocaine induced myocardial ischaemia [9].

Cocaine has also been shown to have caused cerebral infarcts when injected intravenously. Although very rare but it is also implicated in case reports to cause bilateral basal ganglia infarcts [10].

In this case the patient presented with cardiovascular, renal, neurological, and metabolic effects of cocaine overdosage. The etiology of the cardiogenic shock was either myocardial ischaemia or broad complex tachycardia secondary to sodium channel antagonism and/or hyperkalemia.

The decision to undertake coronary angiography was influenced by the recognised complications of cocaine toxicity [10]. After coronary intervention the focus of treatment was hyperkalemia. At this point a trial of IV sodium bicarbonate would have been worthwhile. Once the serum potassium was corrected the rhythm became normal. The important point is that the DC cardioversion did not restore normal sinus rhythm because of the ongoing hyperkalemia. Earlier vigorous treatment of the hyperkalemia might have improved the cardiac output and may have reduced the severity of the multi-organ failure.

We suggest that patients presented to emergency medicine department with cocaine overdosage and electrolyte abnormality should have this corrected as a priority. Untreated hyperkalemia has a high mortality.
